# DNA-PK: gatekeeper for IKKγ/NEMO nucleocytoplasmic shuttling in genotoxic stress-induced NF-kappaB activation

**DOI:** 10.1007/s00018-019-03411-y

**Published:** 2020-01-13

**Authors:** Senad Medunjanin, Maximilian Putzier, Till Nöthen, Sönke Weinert, Thilo Kähne, Blerim Luani, Werner Zuschratter, Ruediger C. Braun-Dullaeus

**Affiliations:** 1 Internal Medicine/Cardiology, Angiology and Pneumology, Magdeburg University, Leipziger Strasse 44, 39120 Magdeburg, Germany; 2 Leibniz Institute for Neurobiology, Magdeburg, Germany; 3 Institute of Experimental Internal Medicine, Magdeburg University, Magdeburg, Germany

**Keywords:** Signal transduction, Inflammation, NF-κB pathway, DNA-PK, IKKγ/NEMO

## Abstract

The transcription factors of the nuclear factor κB (NF-κB) family play a pivotal role in the cellular response to DNA damage. Genotoxic stress-induced activation of NF-κB differs from the classical canonical pathway by shuttling of the NF-κB Essential Modifier (IKKγ/NEMO) subunit through the nucleus. Here, we show that DNA-dependent protein kinase (DNA-PK), an enzyme involved in DNA double-strand break (DSB) repair, triggers the phosphorylation of NEMO by genotoxic stress, thereby enabling shuttling of NEMO through the nucleus with subsequent NF-κB activation. We identified serine 43 of NEMO as a DNA-PK phosphorylation site and point mutation of this serine to alanine led to a complete block of NF-κB activation by ionizing radiation (IR). Blockade of DNA-PK by a specific shRNA or by DNA-PKcs-deficient cells abrogated NEMO entry into the nucleus, as well. Accordingly, SUMOylation of NEMO, a prerequisite of nuclear NEMO, was abolished. Based on these observations, we propose a model in which NEMO phosphorylation by DNA-PK provides the first step in the nucleocytoplasmic trafficking of NEMO.

**Electronic supplementary material:**

The online version of this article (10.1007/s00018-019-03411-y) contains supplementary material, which is available to authorized users.

## Introduction

DNA-dependent protein kinase (DNA-PK) is a serine–threonine protein kinase that is ubiquitously expressed in all mammalian cells. This kinase consists of a catalytic subunit (DNA-PKcs) and two regulatory subunits (Ku70 and Ku80) [1]. DNA-PK is mainly known as a critical component in the nonhomologous end-joining (NHEJ) pathway of double-strand break (DSB) repair in mammalian cells [2]. Furthermore, DNA-PK was proposed to be a molecular sensor for DNA damage that increases DSB repair mechanisms by phosphorylation of several downstream targets [3, 4]. In addition to its role in DNA repair, DNA-PK is involved in signalling pathways other than the DNA repair pathway. For example, the enzyme triggers apoptosis after DNA damage caused by ionizing radiation (IR) [5]. Additionally, it has antiproliferative effects through the phosphorylation of p53 in collaboration with GSK-3 [6].

A central regulator of the immune system is the transcription factor nuclear Factor κB (NF-κB). NF-κB is involved in the regulation of cellular survival, immune responses and inflammation [7]. Two signalling pathways lead to activation of NF-κB, namely, the canonical pathway and the alternative pathway [8, 9]. The canonical NF-κB pathway is mediated by the IKK complex, which consists of IKKα, IKKβ and the regulatory subunit IKK-γ/NF-κB essential modulator (NEMO) [10]. IKKα and IKKβ, but not the IKKγ/NEMO dimer, are catalytic subunits [10, 11]. Although devoid of catalytic activity, IKKγ/NEMO is strictly required for canonical NF-κB activation [12]. In contrast, the alternative pathway plays a pivotal role in the cellular response to DNA damage, such as IR-induced damage [13–15]. This pathway is tightly regulated by NEMO, which shuttles through the nucleus by undergoing different post-translational modifications, such as phosphorylation, SUMOylation and ubiquitination [16–18], before binding to the IKK complex and subsequent NF-κB activation. The DNA damage response is coordinated by members of the phosphoinositide-3-kinase-related protein kinase (PIKK) family composed of ataxia telangiectasia mutated (ATM), ATM and Rad3 related (ATR) and DNA-PK. Others have shown a requirement for NEMO phosphorylation by ATM following DNA damage, which is important for NF-κB activation [16].

Although regulation of NF-κB by DNA-PK had already been reported [19], an assumption due to the observation that IR-induced NF-κB activation in DNA-PK-deficient cells, M059J, was completely blocked [20], but the exact mechanism is not clear. In our study, we identified DNA-PK as an enzyme involved in the first step of NEMO translocation into the nucleus during genotoxic stress.

## Materials and methods

### Cell culture

All culture media and supplements were purchased from PAA Laboratories (Germany). M059K, M059J, HEK293 and NIH3T3 cells and peripheral blood mononuclear cells (PBMNCs) were grown as described [21]. All procedures involving human materials were approved by the local ethics committee of Magdeburg University in compliance with the principles of the Declaration of Helsinki. PBMNCs were isolated as described [21]. Adherent cells were further cultivated for 14 days to obtain monocyte-derived macrophages.

### Plasmids

The full-length cDNA of human NEMO was cloned into pTagGFP-N (Evrogen). Site-directed mutagenesis of NEMO (serine to alanine) was performed using the QuikChange Site-Directed Mutagenesis Kit from Stratagene (La Jolla, CA). The NEMO-S43A and NEMO-S85A mutants were generated by PCR using the wild-type construct as a template. Mutations were verified by DNA sequence analysis. Reporter constructs containing five copies of NF-κB (NF-κB response element) were purchased from Promega (Mannheim, Germany).

### Reagents and antibodies

The following antibodies were used: NF-κB Pathway Sampler Kit (#9936) (New England BioLabs, Frankfurt/Main, Germany), anti-pSer-43-NEMO (Abgent, Heidelberg, Germany), phospho-specific anti-pSer-43-NEMO (Eurogentec, Köln, Germany), anti-Ku70 (#sc-1487), anti-DNA-PKcs (#sc-5282) (Santa Cruz Biotechnology, Heidelberg, Germany), anti-Ku70/Ku80 (Abcam, Cambridge, UK), anti-ubiquitin from DAKO (Hamburg, Germany), anti-IKKγ/NEMO (#I5032), anti-IKKγ/NEMO (#WH0008517M1), anti-β-actin (Sigma-Aldrich, Germany), and anti-UK (Invitrogen, Germany). Recombinant human TNFα was purchased from Miltenyi (Bergisch Gladbach, Germany), and the DNA-PK inhibitor NU7026 was purchased from Calbiochem (La Jolla, CA, USA).

### Luciferase assay

HEK293 cells stably transfected with 5xNF-κB-RE (Promega, Mannheim, Germany) were washed with phosphate-buffered saline (Mg2 + and Ca2 +  free) and lysed in 150 µl/well luciferase cell culture lysis reagent (Promega, Mannheim, Germany). Luciferase assays were performed using the luciferase assay system from Promega, according to the manufacturer’s instructions, and quantified with a luminometer (LB9506, Berthold, Bad Wildbad, Germany).

### Preparation of nuclear extracts

Preparation of nuclear extracts has been described previously [22].

### Immunoprecipitation

Immunoprecipitation was performed as described previously [22]. In addition, for isolation of GFP-tagged proteins, we used the GFP Isolation Kit (#130-091-125), according to the manufacturer’s instructions (Miltenyi, Bergisch Gladbach, Germany).

### Transfection

Transfection of human mononuclear cells with siRNA was performed using Viromer (Lipocalyx, Halle, Germany) according to the manufacturer’s instructions. The siRNA oligonucleotides with 3′-TT overhangs were purchased from MWG-BIOTECH AG (Ebersberg, Germany). The following siRNA sequences were used: siDNA-PKcs 1: 5′- GAUCGCACCUUACUCUGU UTTdTdT-3; siGL-3: 5′- CUUACGCUGAGUACUUCGATTdTdT-3. The concentration of siRNAs was 20 nmol/l during transfection. In most experiments, siRNA transfection was repeated after 24 h.

### In vitro phosphorylation assay

Recombinant human NEMO (Abnova, Heidelberg, Germany) or purified GST-NEMO fusion proteins (wild-type and mutant) were incubated with DNA-PK (Promega, Mannheim, Germany) at 30 °C in a total volume of 30 μl of DNA-PK kinase assay buffer containing 10 μCi of [γ-32P]ATP (5000 Ci/mmol). Phosphoprotein products were detected by PAGE (10% gel).

### Ionizing irradiation

Ionizing irradiation was performed using a gamma-irradiation device from the BIOBEAM 8000 series (Gamma-Service Medical, Leipzig, Germany).

### Staining of cell cultures for STED microscopy

Fixation and staining of the cells for stimulated emission depletion (STED) microscopy have been described previously [23]. Incubation with the primary antibody was performed overnight at 4 °C. The following antibodies were used: anti-Ku70 (#ab 83501) (Abcam, Berlin, Germany) and anti-IKKγ/NEMO (#SAB1404591) (Sigma-Aldrich, Taufkirchen, Germany). The following secondary antibodies were used: anti-rabbit ATTO 647N (dilution 1:200) and anti-mouse Chromeo 494 (dilution 1:50) (Active Motif, La Hulpe, Belgium).

### Confocal microscopy

Multichannel stacks of immunocytochemically stained macrophages were sequentially recorded using a Leica SP8 confocal microscope (Leica Microsystems, Wetzlar) equipped with a 405-nm diode laser, a supercontinuum white light laser source and sensitive hybrid photomultipliers (HYD). Cells were scanned using a 63 × oil immersion objective with a numerical aperture of 1.4 (HCX PL APO CS 63.0 × 1.40 OIL UV). The diameter of the confocal pinhole was set to 95.5 µm (= Airy 1), and images were taken at a 2048 × 2048 pixel resolution with zoom factor 3 and a scan rate of 700 Hz with a line average of 8. Eighteen optical planes were scanned in the axial direction with a step size between two focal planes of 0.3 µm. These settings resulted in an image volume of 61.48 µm (*x*) × 61.48 µm (*y*) × 5.07 µm (*z*) with a voxel size of 30 nm (*x*) × 30 nm (*y*) × 300 nm (*z*) at 8 bit greyscale resolution.

For spectral filtering, the laser microscope was equipped with acoustooptic modulators (AOTF) and an acoustooptical beam splitter (AOBS) that separates the detection beam path from excitation. The fluorophores of the secondary antibodies were sequentially excited with wavelengths of 488 nm, 553 nm and 405 nm for image acquisition of FITC in the spectral range of 503 nm to 531 nm (channel 1), CY3 in the spectral range of 562–593 nm (channel 2) and DAPI in the spectral range of 412–475 nm (channel 3). With this sequential excitation setting, any crosstalk between channels could be excluded.

For determination of colocalization between Ku 70/80 and NEMO in the nucleus and cytoplasm, image stacks were also assessed in the *xz *or *yz* direction. For image improvement and contrast enhancement, we used Huygens deconvolution software (SVI, Hilversum, The Netherlands) and ImageJ (NIH, USA).

### Statistical analysis

Statistical analysis was performed by ANOVA. Post-test multiple comparison was performed by the Bonferroni method. All experiments were independently repeated at least three times.

## Results

### DNA-PK is involved in NF-κB activation

**Fig. 1:**
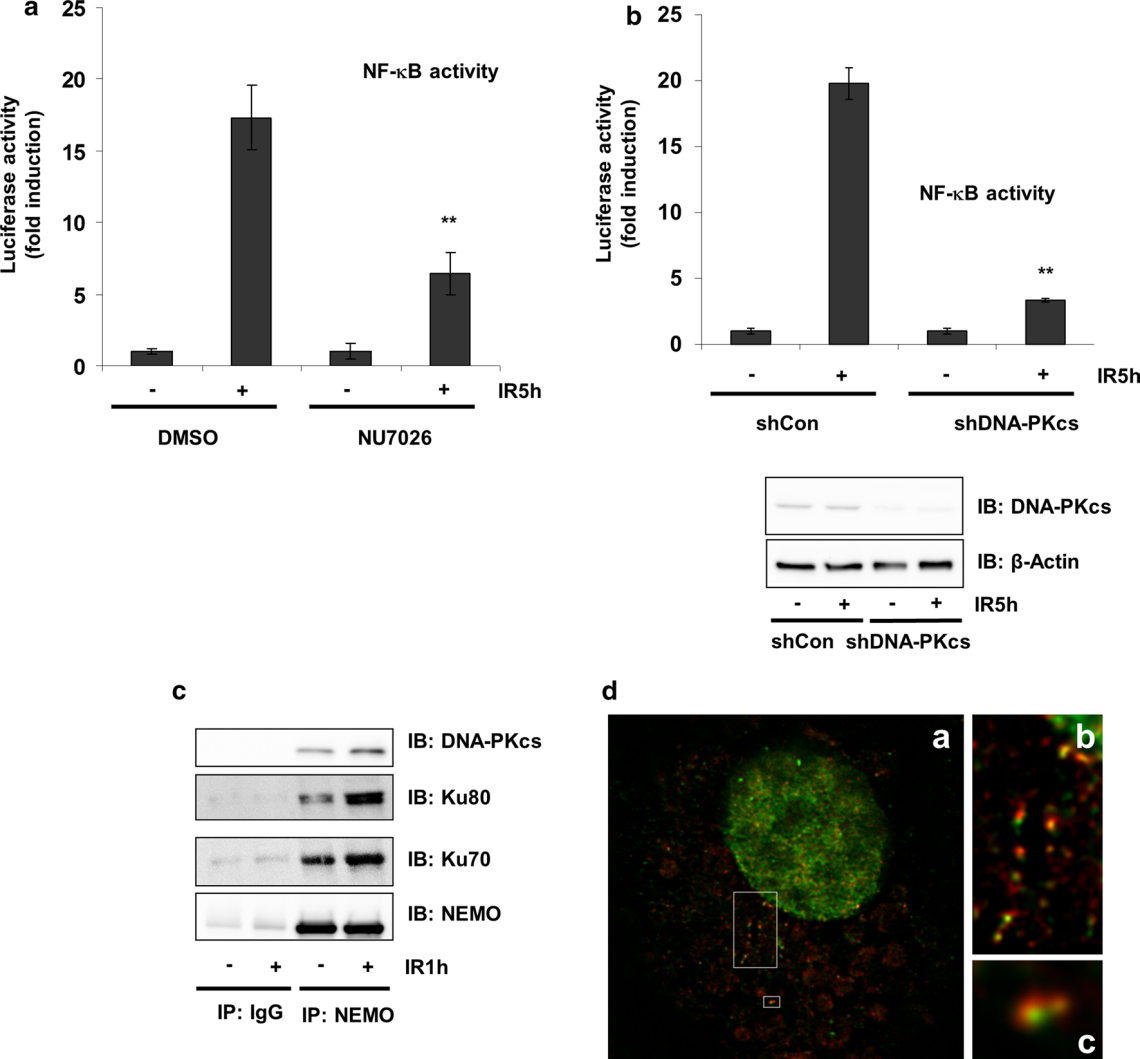
NF-κB DNA-PK is involved in NF-κB activation. **a** After pretreatment with 1 μM Nu7026 for 20 h, cells were exposed to irradiation (IR, 10 Gy) and incubated for 5 h. NF-κB-dependent transactivation was quantified by measuring luciferase activity in cells stably transfected with 5xNF-κB-RE. Data are expressed as a multiple (-fold) of the luciferase activity measured in untreated cells. Error bars represent the S.D. of three experiments (two measurements) (**p < 0.01). **b** HEK293 cells were stably transfected either with control shRNA (sh Ctr) or with shRNA-targeting DNA-PK (sh DNA-PK). Cells were exposed to irradiation (IR, 10 Gy) and incubated for 5 h. NF-κB-dependent gene expression was quantified by measuring luciferase activity. Fold induction is the ratio of stimulated to unstimulated cells (**p < 0.01). **c** CoIP of NEMO and DNA-PK subunits from the lysates of HEK293 cells after exposure of cells to irradiation (IR, 10 Gy) and subsequent incubation for 1 h. **d** HEK293 cells were exposed to irradiation (IR, 10 Gy) and additionally incubated for 1 h. Distribution of endogenous NEMO (red) and Ku70 (green) visualized by immunostaining of HEK293 cells and subsequent STED imaging. The boxed area in (a) is magnified in (b) and (c). While NEMO immunoreactivity was found in “speckles” within the cytoplasm, Ku70 exhibited a dispersed distribution throughout the cytoplasm, where it was associated with NEMO

HEK293 cells stably transfected with a luciferase reporter gene under the control of an NF-κB response element were irradiated after treatment with or without the DNA-PK inhibitor NU7026 [24]. IR resulted in an ~ 20-fold induction in luciferase activity, which was significantly reduced by NU7026 treatment (Fig. 1a). Similar results were obtained using shDNA-PKcs (Fig. 1b). Thereafter, we examined whether NEMO and DNA-PK can physically interact by immunoprecipitation of endogenous NEMO and analysis of the immune complexes for the presence of NEMO or DNA-PK subunits. Association of DNA-PK subunits and NEMO was observed in untreated cells, which increased after IR treatment (Fig. 1c). The interaction of either was confirmed by super-resolution STED microscopy, visualizing colocalization of NEMO and Ku70 mainly within the cytoplasm but close to the nucleus in HEK293 cells (Fig. 1d).

### DNA-PK phosphorylates NEMO at Ser43

**Fig. 2:**
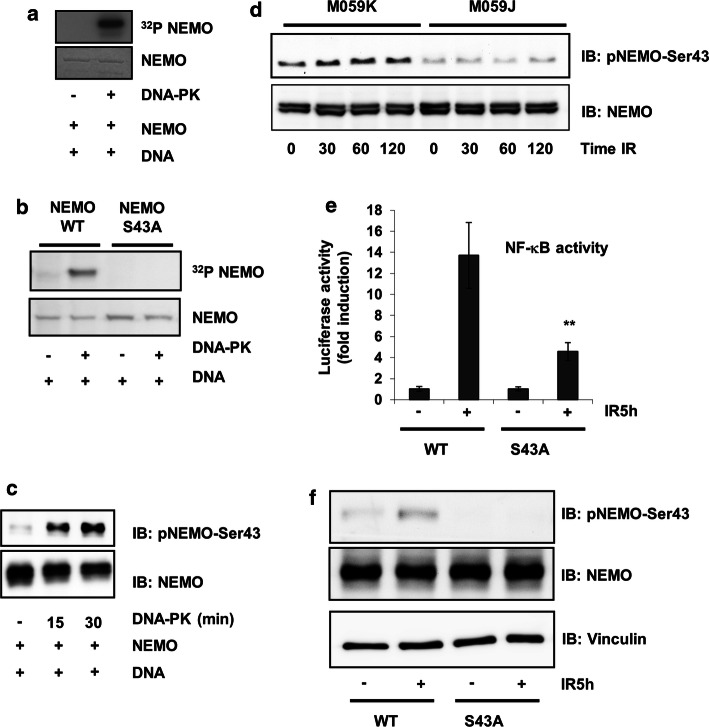
IKKγ/NEMO phosphorylation is dependent on DNA-PK. **a** In vitro kinase assay using recombinant human NEMO (1 μg) as a substrate for DNA-PK (0.01 µg). **b** In vitro kinase assay using GST-isolated NEMO (1 μg) as a substrate for DNA-PK (0.01 µg). **c** In vitro kinase assay using recombinant human NEMO (0.5 μg) as a substrate for DNA-PK (0.01 µg), incubated for different time intervals as indicated. **d** M059K and M059J cells were exposed to ionizing radiation (IR, 10 Gy) for the indicated time intervals, after which the protein extracts were subjected to immunoblot analysis with antibodies to NEMO or phospho-NEMO-Ser-43. **e** HEK293 cells were transfected with expression vectors carrying wild-type and mutant NEMO. Cells were exposed to irradiation (IR, 10 Gy) and incubated for 5 h. NF-κB-dependent gene expression was quantified by measuring luciferase activity. Fold induction is the ratio of stimulated to unstimulated cells (**p < 0.01). **f** Cell lysates from (**e**) were immunoblotted with the indicated antibodies

Because phosphorylation is an important step in NEMO regulation, we investigated whether NEMO is a target of DNA-PK. Using recombinant human NEMO (rhNEMO) and DNA-PK, we induced in vitro NEMO phosphorylation by DNA-PK (Fig. 2a). To identify the sites phosphorylated by DNA-PK, we performed several mass spectrometric analyses of NEMO immunoprecipitates after treatment of cells with the DNA-PK inhibitor NU7026 (example shown in Suppl. Fig. 1a, b) and observed reduced phosphorylation of NEMO at Ser43 (not shown). Furthermore, reduced SUMOylation of NEMO in cells treated with the DNA-PK inhibitor was observed. These results implied that phosphorylation of Ser43 is a prerequisite for NEMO SUMOlyation and further NF-κB activation. Our findings from the mass spectrometric analyses were confirmed in an in vitro phosphorylation assay of wild-type NEMO and the NEMO mutant in which this serine was mutated to alanine (Fig. 2b). The phosphorylation of NEMO was further investigated by assessing in vitro phosphorylation of recombinant NEMO using phospho-specific antibodies. Indeed, DNA-PK could phosphorylate serine 43 in an in vitro kinase assay (Fig. 2c). The phosphorylation state of the Ser43 residue was further studied in lysates of M059K and DNA-PKcs-deficient M059J human cell lines. Commensurate with the activation of DNA-PK by IR, we found an increase in NEMO phosphorylation in a time-dependent manner in M059K cells (Fig. 2d). This phosphorylation was abolished in DNA-PK-deficient M059J cells (Fig. 2d).

We then studied the impact of Ser43 phosphorylation on NF-κB transcriptional activity. For this purpose, NEMO-WT and a NEMO-Ser43A mutant were overexpressed in HEK293 cells stably transfected with a 5xNF-κB response element, and NF-κB activity was determined. Mutation of Ser43 of NEMO reduced the activation of NF-κB, indicating that Ser43 is required for full transcriptional activation of NF-κB by genotoxic stress, such as IR (Fig. 2e). Furthermore, using a phospho-specific antibody recognizing Ser43, we confirmed that irradiation resulted in phosphorylation of this site, which was abolished when the mutant of NEMO, where serine was changed to alanine, was overexpressed (Fig. 2f).

### DNA-PK-mediated phosphorylation induces NEMO nuclear translocation

**Fig. 3:**
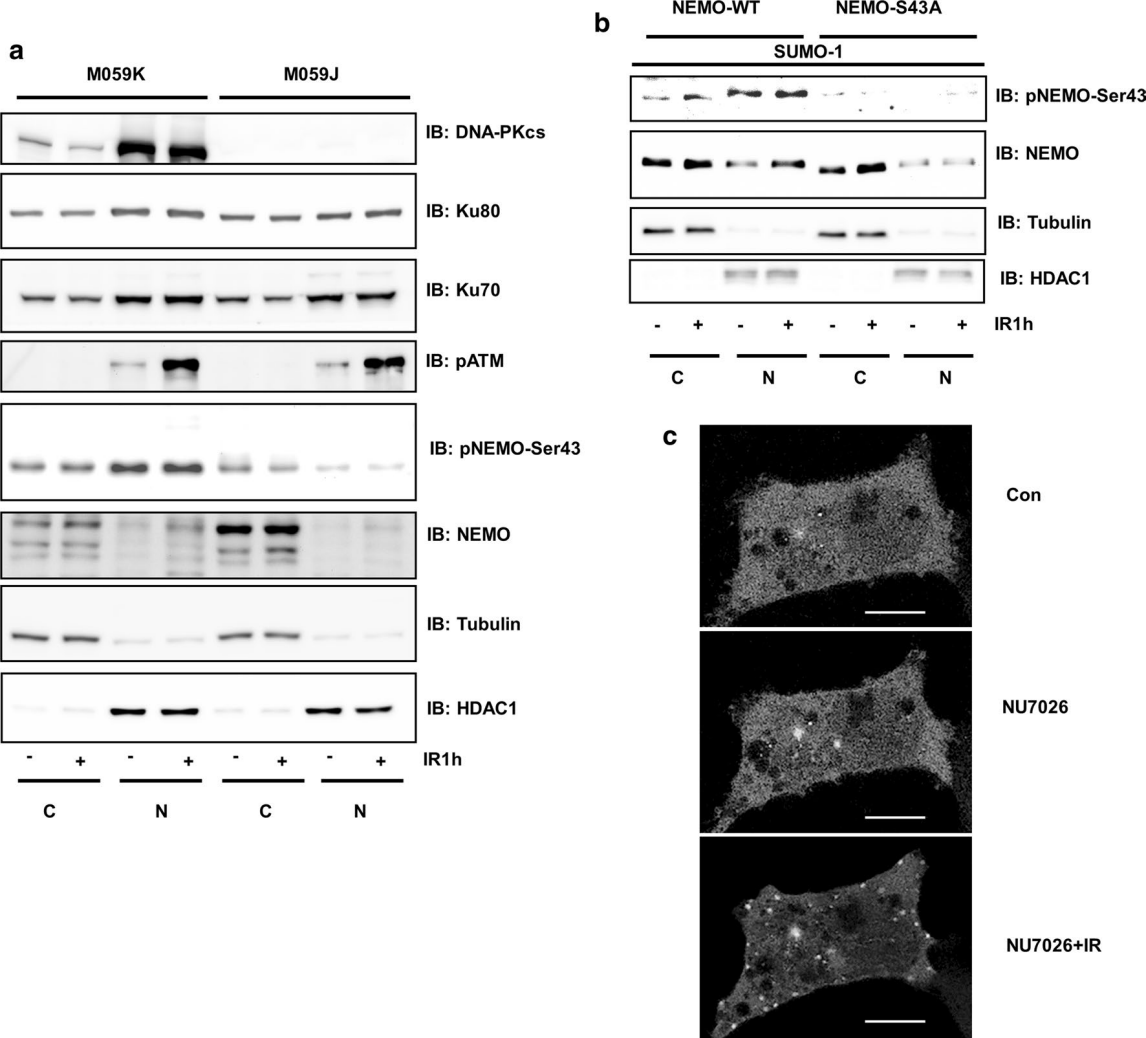
DNA-PK-mediated phosphorylation induces NEMO nuclear translocation**. a** M059J and M059K cells were exposed to IR (10 Gy) and incubated for 1 h. Cells were fractionated; nuclear (N) and cytoplasmic (C) proteins were analysed by immunoblotting with antibodies as indicated. **b** HEK293 cells were cotransfected with a SUMO-1 plasmid and either wild-type NEMO or mutant NEMO. After IR treatment (20 Gy) for 1 h, cells were fractionated; nuclear (N) and cytoplasmic (C) proteins were analysed by immunoblotting with the indicated antibodies. **c** HEK293 cells were transfected with wild-type NEMO-GFP. The indicated cells were pretreated with NU7026 (1 µM) followed by IR exposure (IR, 10 Gy). Confocal microscopy was performed over a time period of 2 h. The scale bar indicates 10 µm

The potential involvement of DNA-PK in NEMO regulation was further studied in M059K and M059J cells. Since the prerequisite for genotoxic stress-activated NF-κB is the translocation of NEMO from the cytoplasm to the nucleus [8], we performed fractionation of cells and tested the phosphorylation and localization of NEMO. Phosphorylation of ATM was used as a positive control for IR-induced phosphorylation within the nucleus (Fig. 3a). We observed strong nuclear phosphorylation of NEMO at Ser43 in M059K cells. This nuclear phosphorylation was almost lost in M059J cells (Fig. 3a). Instead, increased expression of NEMO within the cytoplasm of M059J cells was observed. These results were confirmed by overexpression of wild-type and mutant NEMO in HEK293 cells (Fig. 3b). Intriguingly, overexpression of GFP-tagged NEMO and simultaneous blockade of DNA-PK resulted in a dramatic increase in punctate fluorescence [25] within the cytoplasm of HEK293 cells, as shown by confocal microscopy, confirming that inhibition of DNA-PK indeed increases NEMO accumulation within the cytoplasm (Fig. 3c). In addition, this result was confirmed by overexpression of mutant NEMO in HEK293 cells (Suppl. Fig. 2a).

### Interaction of DNA-PK with NEMO in macrophages

**Fig. 4:**
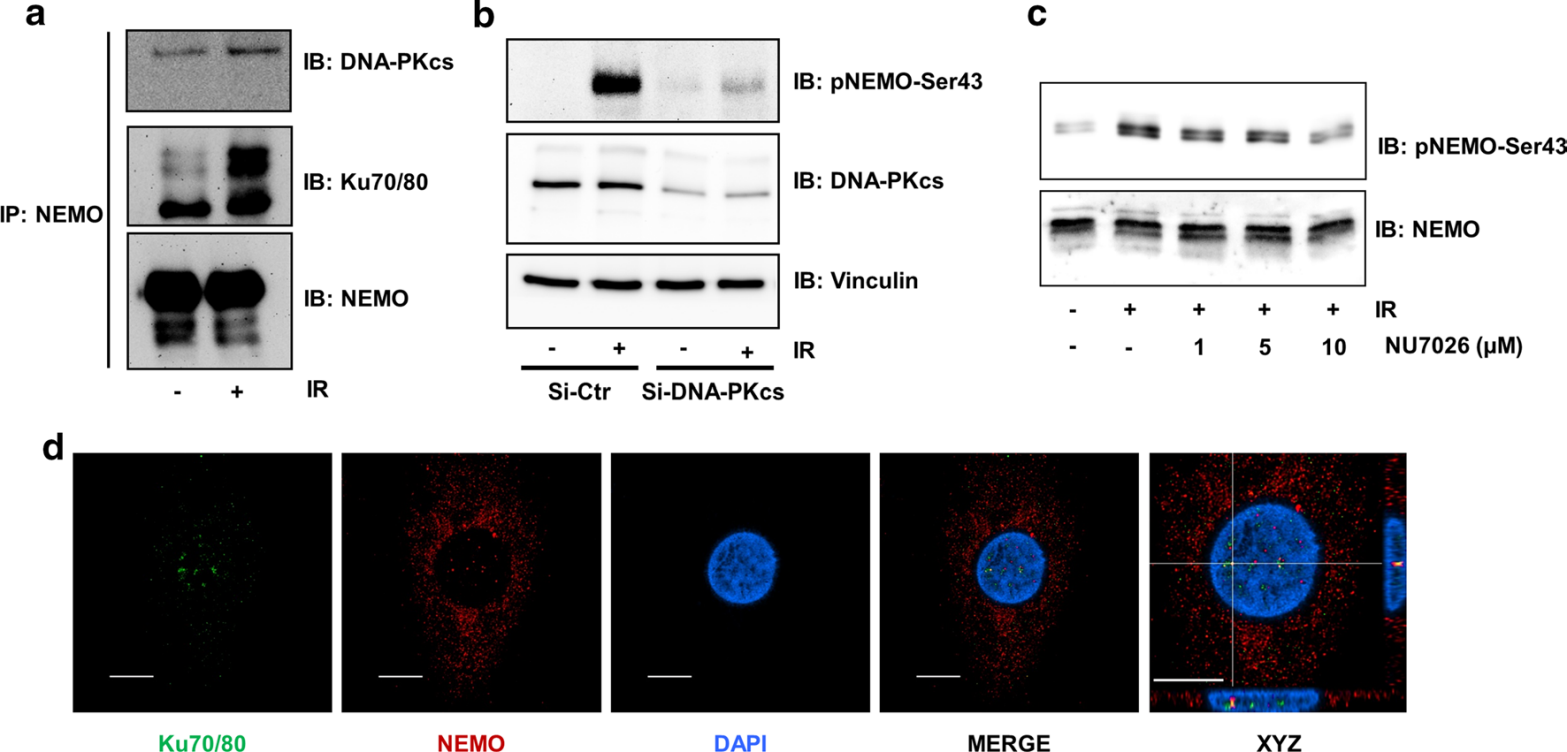
DNA-PK/IKKγ interaction in primary macrophages**. a** CoIP of NEMO and DNA-PK subunits from the lysates of macrophages after exposure of cells to irradiation (IR, 5 Gy) and subsequent incubation for 1 h. **b** Human primary macrophages were transfected with either GL3 control siRNA (siCtr) or siRNA targeting the DNA-PK subunit (siDNA-PKcs). After pretreatment with siRNA for 24 h, cells were exposed to IR (5 Gy), and lysates were analysed by immunoblotting (IB) with specific antibodies as indicated. **c** Human primary macrophages were incubated in the presence of different doses of the DNA-PK inhibitor NU7026 for 2 h. After exposure to irradiation (IR, 5 Gy), lysates were analysed by IB with specific antibodies as indicated. **d** Immunofluorescence staining of macrophages with antibodies against Ku 70/80 (green) and NEMO (red) and nuclear staining with DAPI (blue). In the merged image, the magnified region of interest (right picture) and particularly the xz and yz projections of individual clusters of Ku 70/80 and NEMO were localized very close to each other in the euchromatin of the nucleus. Scale bar indicates 10 µm

Different cell types and tissues show different sensitivities towards NF-κB activation by IR. Therefore, we confirmed that the interaction of DNA-PK with NEMO also occurs in primary cells. For this purpose, peripheral blood monocytes were isolated and matured towards macrophages. After lysis of the cells and immunoprecipitation of NEMO, a strong interaction of NEMO with DNA-PK subunits was observed, which increased after IR exposure (Fig. 4a). Furthermore, we tested the phosphorylation of NEMO at Ser43 after exposure of macrophages to IR and observed strong phosphorylation at Ser43, which was reduced using siRNA to block DNA-PKcs levels (Fig. 4b). Similarly, NEMO phosphorylation was sensitive to DNA-PK inhibition by the specific DNA-PK inhibitor NU7026 (Fig. 4c). The interaction was further confirmed using confocal microscopy, which showed interaction of NEMO with the DNA-PK subunit Ku70 within the nuclei of macrophages (Fig. 4d).

### DNA-PK/NEMO interaction modulates the alternative NF-κB pathway

**Fig. 5:**
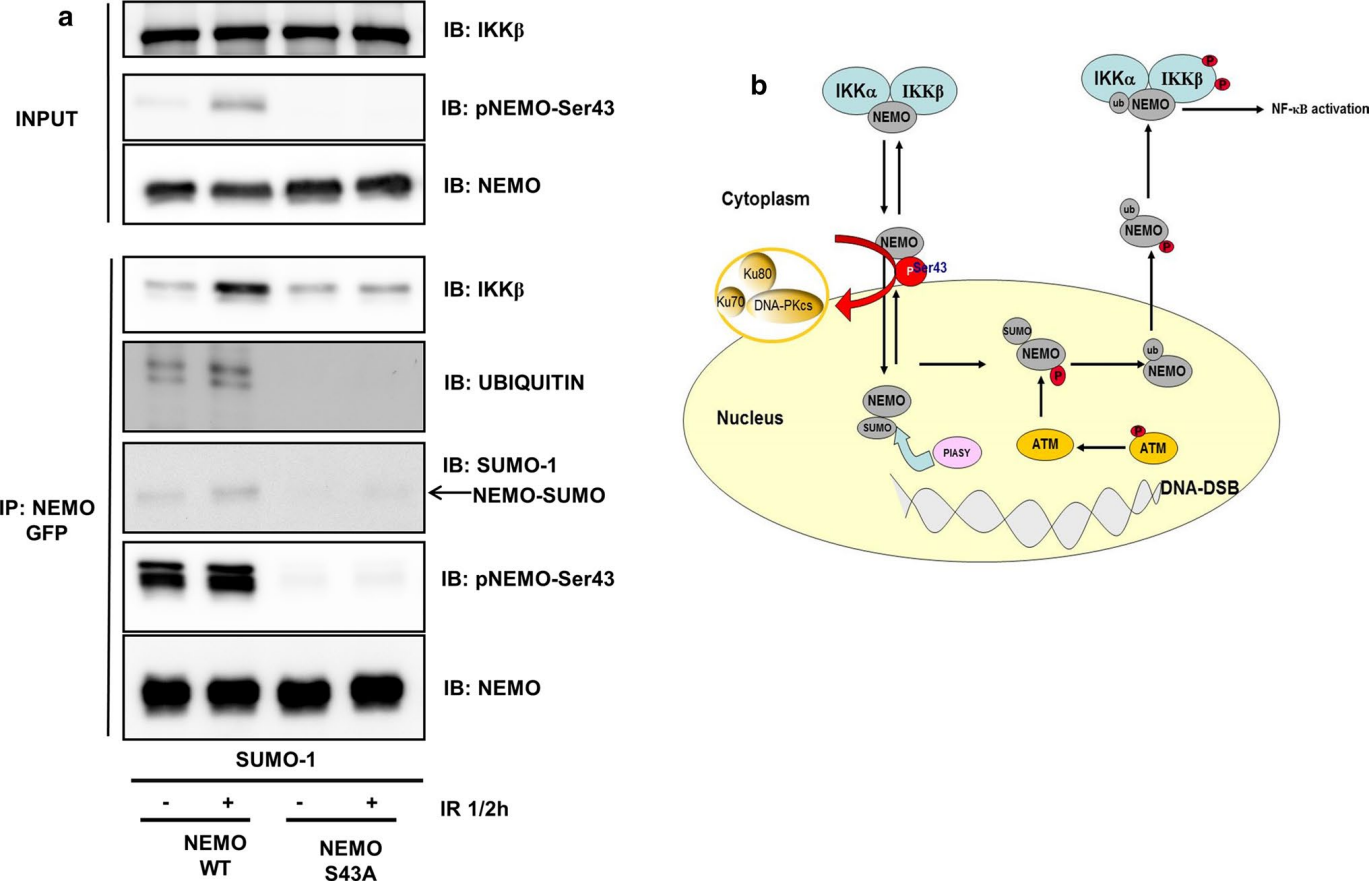
DNA-PK/IKKγ interaction modulates NEMO nuclear translocation. **a** HEK293 cells were cotransfected with a SUMO-1 plasmid and either wild-type NEMO or mutant NEMO. After exposure to irradiation (IR, 10 Gy), the cells were additionally incubated for 30 min. The lysates were immunoprecipitated (IP) with anti-GFP, followed by immunoblotting (IB) with the indicated antibodies. **b** Proposed model of DNA-PK function in NF-κB activation by genotoxic stress: Under genotoxic stress, DNA-PK phosphorylates NEMO at Ser43, which allows entry of the protein into the nucleus; then, the SUMO ligase PIASy and the kinase ataxia telangiectasia mutated (ATM) SUMOylate and phosphorylate nuclear IKKγ (NEMO), which results in ubiquitination of NEMO, expulsion from the nucleus and activation of the cytoplasmatic IKK complex [41, 42]

The interaction between DNA-PK and NEMO was further examined in the context of previous reports. Several groups have shown that cytoplasmic SUMOylation, phosphorylation and ubiquitination of NEMO play a critical role in the genotoxic activation of NF-κB [16, 17, 26]. Therefore, we performed cotransfection of NEMO and the mutant NEMO with a SUMO-1 plasmid (Fig. 5a). We performed immunoprecipitation of NEMO with a GFP tag and tested well-assessed checkpoints within the stress-induced pathway of NF-κB activation. Immunoblotting of the immunoprecipitates revealed increased ubiquitination of wild-type NEMO after exposure of the cells to IR (Fig. 5a). This ubiquitination was completely abolished in the mutant NEMO (Fig. 5a). Furthermore, NEMO SUMOylation and binding to IKKβ were both reduced in the mutant NEMO compared to wild-type NEMO (Fig. 5a). In addition, we tested the phosphorylation of NEMO at Ser43 by overexpression of a previously reported Ser85 mutant of NEMO [16] and observed slightly reduced phosphorylation of NEMO (Suppl. Fig. 2b).

## Discussion

Previous studies have demonstrated the involvement of DNA-PK in NF-κB activation. For example, the Ku subunit of DNA-PK can interact with the NF-κB p50 promoter in gastric cancer cells and, in this way, acts as a positive regulator of p50 protein expression [27]. Additionally, DNA-PK can modulate the NF-κB subunit p65 [28]. Furthermore, activation of the alternative genotoxic stress-induced pathway through DNA-PK has also been proposed because IR-triggered NF-κB activation was completely abolished in DNA-PK-deficient M059J cells [20]. However, the mechanism remains unclear. A central protein in the genotoxic activation of NF-ĸB is NEMO, which takes an alternative route through the nucleus before binding to the IKK complex and activating NF-κB [29]. In this report, we identified NEMO as a new DNA-PK substrate that is phosphorylated at Ser43 located within the N-terminal domain. This serine residue is involved in the control of NF-κB activation by genotoxic stress. We propose a model in which DNA-PK serves as a gatekeeper for NEMO to enter the nucleus, as abrogation of the Ser43 phosphorylation site results in accumulation of the protein within the cytoplasm and reduced NEMO phosphorylation, SUMOylation and ubiquitination, the prerequisites of nuclear NEMO shuttling (Fig. 5b).

We were surprised to identify Ser43 as a DNA-PK phosphorylation site because DNA-PK usually phosphorylates nuclear proteins at serine or threonine residues followed by glutamic acid [3]. However, a previous report showed that DNA-PK, in response to DNA damage, phosphorylates cytoplasmic GOLPH3 even when glutamic acid is absent [30]. A study describing phosphorylation of Ser43 in NEMO by IKKβ lacks a functional explanation regarding the importance of Ser43 in NEMO for NF-kB activation [31]. Previously, we identified GSK-3β as a kinase that phosphorylates Ser8, 17, and 31 of NEMO during TNFα-induced NF-κB activation [23]. In this study, Ser43 was also found to be phosphorylated. However, overexpression of a Ser43 mutant had no effect on TNFα-induced NF-κB activity, excluding a significant involvement in the canonical NF-κB activation pathway. This observation is concordant with a previous report demonstrating a reduction in IR-induced NF-κB activation in DNA-PKcs-deficient cells, while the response to TNFα was not impaired [20].

Endogenous NEMO is localized predominantly in the cytoplasm. However, following exposure to DNA damage, NEMO translocates to the nucleus [16]. The mechanism underlying this first step of its translocation is not clear, since NEMO lacks a classical nuclear localization signal that would be recognized by importins [32, 33]. However, a recent report by Hwang and colleagues demonstrated that NEMO interacts with the importin β family member IPO3 and translocates to the nucleus [34]. At present, we can only speculate but favour a model in which the Ku70/NEMO interaction modulates NEMO translocation to the nucleus. This assumption is based on the established role of Ku70 in shuttling, for example, in *Rickettsia conorii* [35], and on the nuclear translocation of Ku70 upon exposure of cells to DNA damage [36–38]. How exactly Ku70 can be utilized by cytosolic NEMO to gain entry into the nucleus needs further investigation. Ku70 and NEMO may associate with the nuclear localization receptor importin-α, which enables nuclear translocation [39]. In a recent study [40], phosphorylation of an S/T-P-S/T domain operated as a general nuclear translocation signal, as is the case for Ser43 in NEMO. Therefore, this site may be directly responsible for NEMO nuclear localization. Genotoxic activation of NF-κB requires a series of nuclear post-translational modifications of NEMO that are critical for NF-κB activation following genotoxic stress (reviewed in [29]). N-terminal fusion of NEMO with SUMO-1 targeted NEMO from the cytoplasm to the nucleus with subsequent ubiquitination of NEMO [16, 17]. Subsequent work has identified PIASy as the major SUMO E3 ligase for NEMO that is required for NF-κB activation by multiple genotoxic agents [26]. Interestingly, this report shows the nuclear interaction of PIASy and NEMO, postulating nuclear NEMO SUMOylation. The time course analysis suggested that SUMOylation of NEMO occurs earlier and transiently before ubiquitination of NEMO is observed [17]. The induction of DNA DSBs leads to phosphorylation of NEMO by ATM, and this event seems to occur after SUMOylation and before ubiquitination of NEMO [29]. In our experiments, we observed not only a reduction in NF-κB activation by overexpression of mutant NEMO but also a reduction in NEMO SUMOylation. Future experiments including the fusion of SUMO-1 with the NEMO mutant will explore the significance of NEMO phosphorylation for NEMO SUMOylation and whether SUMOylated NEMO is able to overcome the inability of the NEMO S43A mutant to translocate to the nucleus.

In summary, our data imply a gatekeeper function of DNA-PK for the entry of NEMO into the nucleus under genotoxic stress. This novel mechanism of NEMO regulation by DNA-PK highlights the role of the post-translational modifications of NEMO in NF-κB activation.

Activation of the genotoxic stress-induced NF-κB pathway was completely abolished in DNA-PK-deficient M059J cells.

## Electronic supplementary material

Below is the link to the electronic supplementary material.

**a** HEK293 cells were transfected with either a GFP control plasmid or wild-type NEMO-GFP. After 2 h of pretreatment with NU7026 (1 µmol/l) and exposure to irradiation (IR, 10 Gy), the cells were additionally incubated for 1 h. Lysates were immunoprecipitated (IP) with anti-GFP, followed by MS analysis. **b** 5MS/MS spectrum of the NEMO peptide containing phosphorylated Ser43 in the sample after radiation. m/z = 690.9957, z = 3, mass error = 2.4 ppm, probability score *p* = 4.5 E-6 (TIF 743 kb)

**a** HEK293 cells were transfected with wild-type or mutant NEMO-GFP. The indicated cells were exposed to IR (IR, 10 Gy). Confocal microscopy was performed over a time period of 2 h. The scale bar indicates 10 µm. **b** HEK293 cells were cotransfected with a SUMO-1 plasmid and either wild-type NEMO or mutant NEMO. After IR treatment (10 Gy) for 1 h, proteins were analysed by immunoblotting with the indicated antibodies (TIF 1147 kb)

## Abbreviations

IKKγ/NEMO: NF-B essential modifier
DNA-PK: DNA-dependent protein kinase
RE: Response element
IR: Ionizing radiation
