# Supplementary figures and images for: DNA-PK: gatekeeper for IKKγ/NEMO nucleocytoplasmic shuttling in genotoxic stress-induced NF-kappaB activation
Source: Cell Mol Life Sci. 2020 Jan 13;77(20):4133–42. doi: 10.1007/s00018-019-03411-y (PMC7532968; doi:10.1007/s00018-019-03411-y)

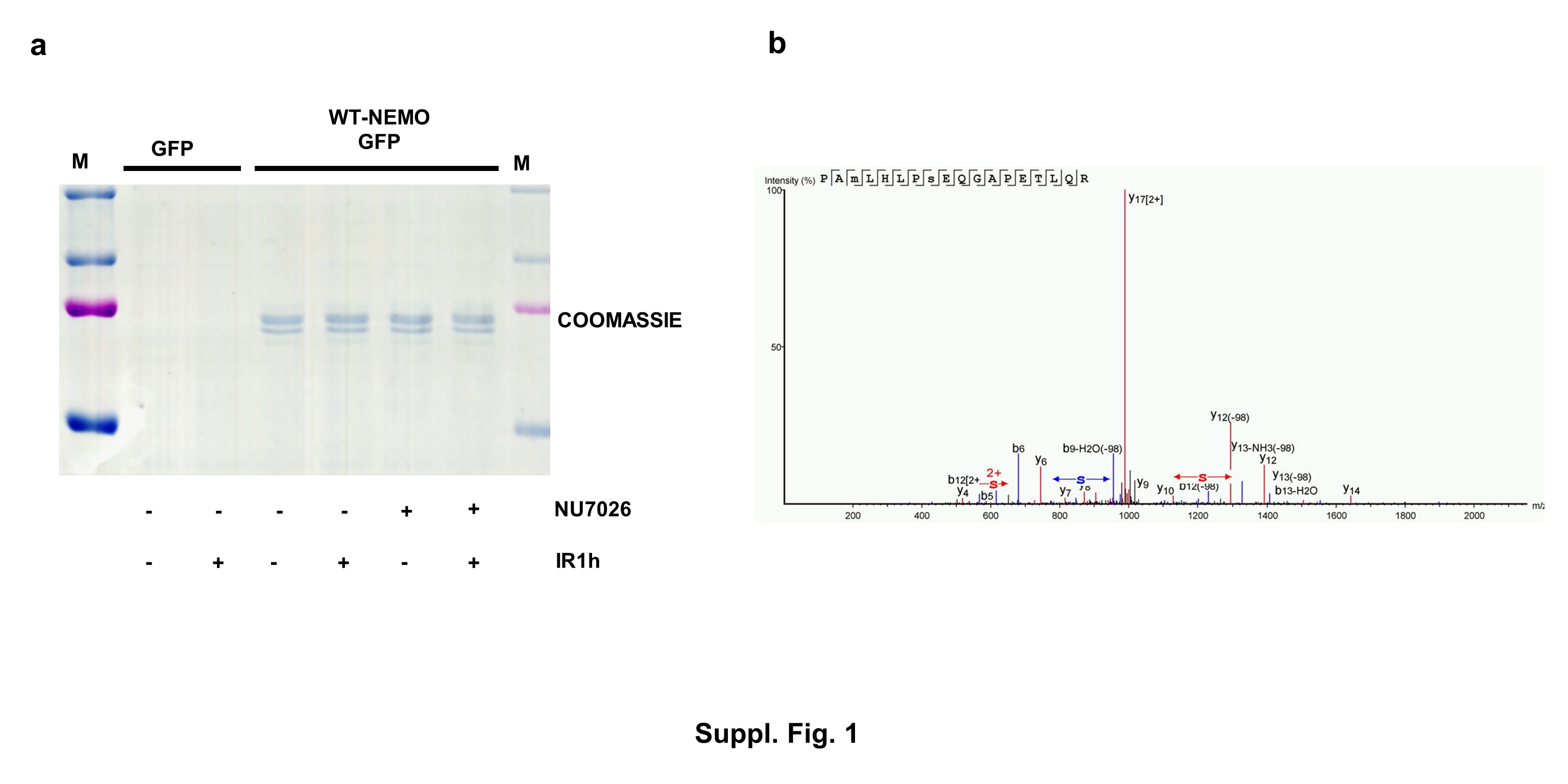

Supplement: Supplementary file 1 — a HEK293 cells were transfected with either a GFP control plasmid or wild-type NEMO-GFP. After 2 h of pretreatment with NU7026 (1 µmol/l) and exposure to irradiation (IR, 10 Gy), the cells were additionally incubated for 1 h. Lysates were immunoprecipitated (IP) with anti-GFP, followed by MS analysis. b 5MS/MS spectrum of the NEMO peptide containing phosphorylated Ser43 in the sample after radiation. m/z = 690.9957, z = 3, mass error = 2.4 ppm, probability score p = 4.5 E-6 (TIF 743 kb) [file 18_2019_3411_MOESM1_ESM.tif]

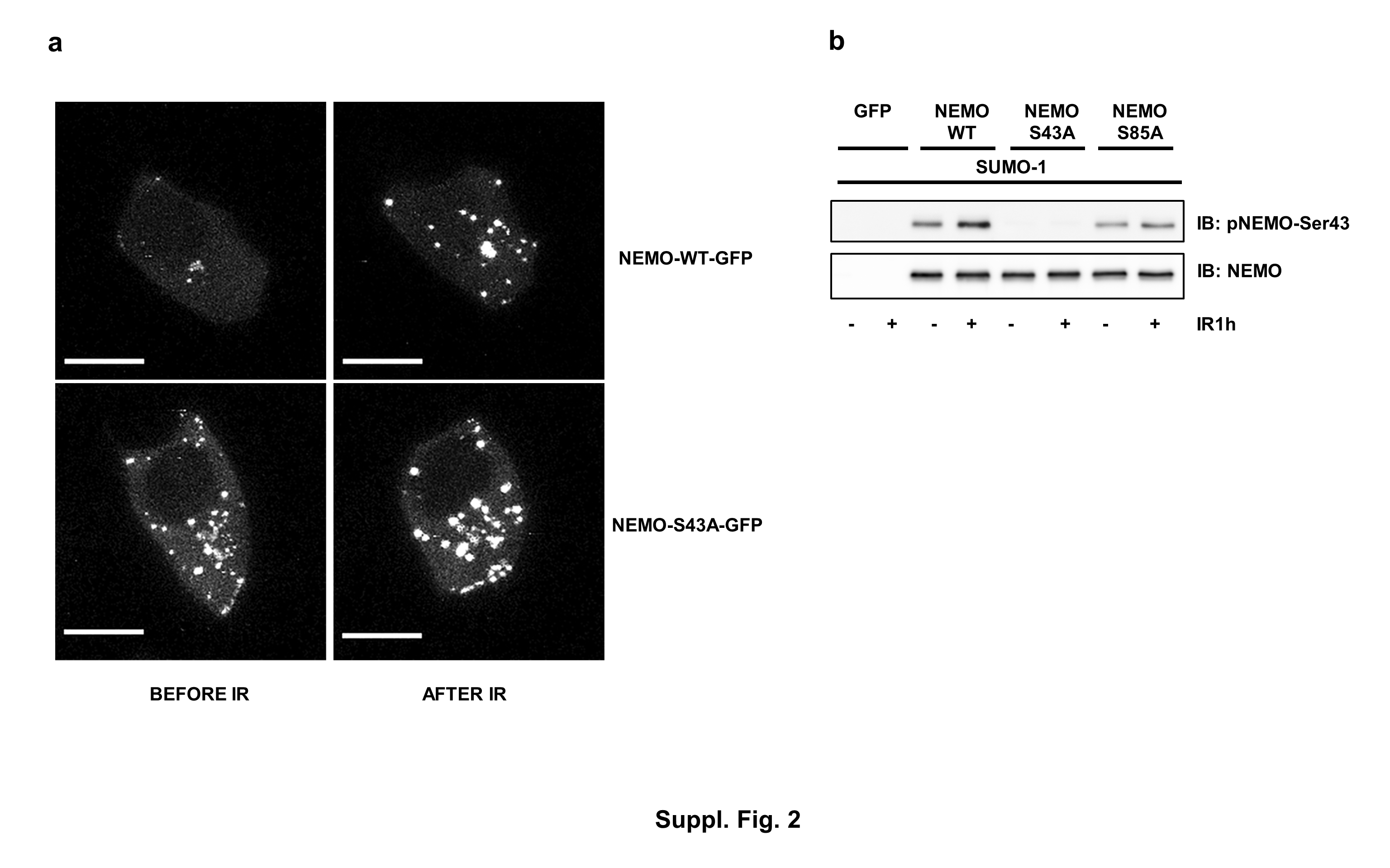

Supplement: Supplementary file 2 — a HEK293 cells were transfected with wild-type or mutant NEMO-GFP. The indicated cells were exposed to IR (IR, 10 Gy). Confocal microscopy was performed over a time period of 2 h. The scale bar indicates 10 µm. b HEK293 cells were cotransfected with a SUMO-1 plasmid and either wild-type NEMO or mutant NEMO. After IR treatment (10 Gy) for 1 h, proteins were analysed by immunoblotting with the indicated antibodies (TIF 1147 kb) [file 18_2019_3411_MOESM2_ESM.tif]
